# Haploidentical and MHC-identical cells exhibit broadly similar immune reconstitution kinetics, with some compartment-specific differences, following hematopoietic cell transplantation in a murine model of severe combined immunodeficiency

**DOI:** 10.3389/fimmu.2026.1960755

**Published:** 2026-10-22

**Authors:** Isabel Hidalgo, Maria Björklund, Cornelis Jan Pronk

**Affiliations:** 1 Medical Faculty, Division of Molecular Hematology, Department of Laboratory Medicine, Lund University, Lund, Sweden; 2 Medical Faculty, Wallenberg Center for Molecular Medicine and Lund Stem Cell Center, Lund University, Lund, Sweden; 3 Childhood Cancer Centre, Skåne University Hospital, Lund, Sweden

**Keywords:** chimerism, haploidentical, hematopoietic cell transplantation, immune reconstitution, lymphoid, mouse model, myeloid, SCID

## Abstract

**Introduction:**

Immune reconstitution following HLA-haploidentical hematopoietic cell transplantation (Haplo-HCT) is often delayed compared with HLA-identical transplantation, which frequently poses clinical challenges. The reasons for these differences in reconstitution kinetics remain poorly understood, and direct comparisons between the two settings are difficult to perform in the clinic.

**Methods:**

We therefore generated a mouse model of Haplo-HCT by crossing C57B6 and BALB/c mice to obtain C6BF1 offspring, and transplanted equal numbers of T- and B-cell-depleted bone marrow cells from either C6BF1 (haploidentical) or C57B6xB6SJL (identical, syngeneic) donors into conditioned, immunodeficient Rag1-deficient recipients that cannot generate autologous mature B or T cells. Immune reconstitution was monitored every 2-4 weeks by blood and immune-cell counts, immunoglobulin production, and T- and B-cell rearrangement activity (TREC/KREC), and the response to viral inflammation was assessed by poly(I:C) injections, with mice analyzed up to 16 weeks after transplantation.

**Results:**

Haploidentically transplanted mice showed higher early chimerism of myeloid and NK cells than identically transplanted mice at 8 weeks, with chimerism no longer significantly different by 16 weeks. Overall B- and T-cell production showed no significant differences between the two groups (apart from a tendency toward lower CD8 T-cell numbers in the haploidentical setting), immunoglobulin recovery was faster after Haplo-HCT, and TREC recovery kinetics showed no significant differences. Inflammatory responses to poly(I:C) showed no significant differences between the two settings in both settings. Overall, Haplo-HCT reconstitution was not found to be inferior to MHC-identical HCT in this model, with several parameters favouring the haploidentical setting, and the higher early chimerism may reflect superior replenishment of haploidentical myeloid and NK cells or cytotoxic activity of haploidentical T cells against residual host cells.

**Discussion:**

These findings indicate that haploidentical grafts are not intrinsically inferior for immune reconstitution in this model and suggest that the delayed reconstitution observed clinically after Haplo-HCT more likely reflects differences in conditioning and graft manipulation than the haploidentical nature of the transplanted cells.

## Introduction

Allogeneic hematopoietic cell transplantation (HCT) is a treatment modality used to fully reestablish the hematopoietic system, typically following myelo- and lymphoablative conditioning therapy. HCT may be required in pathophysiological conditions such as primary immunodeficiencies (1), non-malignant blood diseases (2, 3) or after aggressive radiotherapy- and/or chemotherapy-based treatments used to eliminate various hematological cancers, such as leukemia (4, 5). Ideally, an HLA-identical (or syngeneic) sibling is used, followed in order of preference by an HLA-matched unrelated donor (MUD), to avoid complications arising from graft rejection or graft-versus-host disease (GvHD). Because an identical sibling is unavailable for approximately 70% of patients, and identifying a MUD is not always possible or sometimes requires a longer process than desired, alternative sources of hematopoietic cells have emerged. In recent years, haploidentical donors, who are partially HLA-matched (approximately 50%) to the recipient and are usually the patient’s parents, children, or siblings, have been used as a valuable alternative. This transplantation modality offers the practical advantage that such donors are often close to the recipient and readily available, which reduces both the time needed to search for a donor and the associated cost, an important factor especially in developing countries. In addition, therapeutic benefits can be gained from haploidentical transplantation (Haplo-HCT), as the proximity of the donor allows the transplantation to be repeated if needed and enables the later administration of donor lymphocyte infusions (DLI). DLI offers the potential advantage of exploiting the 50% mismatch to target, for example, minimal residual disease (MRD) after aggressive cancer ablation, thereby achieving a desired graft-versus-tumor (GvT) effect and preventing relapse. On the other hand, to minimize the risk of graft rejection and GvHD, haploidentical transplantation requires both conditioning of the recipient (ranging from reduced-intensity to myeloablative regimens) and immunosuppression of the donor cells. The latter is often achieved *in vivo* by administration of antithymocyte globulin (ATG) or high doses of post-transplant cyclophosphamide (PTCy) (6), or ex vivo by depletion of T (especially αβ), B, or both cell types to avoid alloreactivity (7–9). Because the donor cells are lymphoid-depleted, immune reconstitution is slower, and these patients often show a high incidence of complications related to opportunistic viral or fungal infections (10) or viral reactivation, compared with recipients of fully HLA-compatible cells. The tempo and quality of immune reconstitution are themselves important determinants of clinical outcome after allogeneic HCT: early CD4+ T-cell recovery predicts survival after acute graft-versus-host disease (11), CD4+ and B-cell recovery was associated with lower risk of graft-versus-host disease and relapse centers (12), and the recovery of recent thymic emigrants influences outcomes after HCT for inborn errors of immunity (13). From clinical experience, however, it is not obvious whether this delayed reconstitution is due to the fact that haploidentical cells are transplanted or whether it relates more to differences in pre-transplant conditioning regimens and graft manipulation between donor types (14, 15). Because such factors are difficult to disentangle in clinical studies, dedicated murine studies are required to address this question directly.

Therefore, in this study we used a mouse model to address whether the kinetics of immune reconstitution and cellular performance are comparable between syngeneic and haploidentical TBD bone marrow cells transplanted under similar conditions. In recent years, haploidentical murine models have been generated and used to study their performance in different contexts, such as immunodeficiencies or autoimmune diseases (16, 17), to study their potential for a GvT effect (18–20), and to assess the efficacy of different conditioning regimens (21, 22). However, our model is the first in which a detailed quantitative and functional characterization of the kinetics of immune reconstitution from TBD cells is directly compared between these two donor cell sources. Furthermore, by using Rag1-deficient mice, we developed a system that allows the lymphocyte development derived from the donor cells to be tracked specifically, without interference from the recipient’s residual, autologous cells, because these mice cannot generate mature B or T cells. This work thus contributes to a better understanding of immune reconstitution by donor cells transplanted for either primary immunodeficiencies or following aggressive ablative treatments. More broadly, it provides an immunological framework for dissecting how donor-intrinsic properties shape the kinetics of innate and adaptive reconstitution, independently of the confounding variables present in the clinical setting.

## Materials and methods

### Mice strains

Wild type mice from C57Bl6 (H2^b^/CD45.2), B6SJL (H2^b^/CD45.1) and BALB/c (H2^d^/CD45.2) strains were bred in-house at the Center of Comparative Medicine (CCM) of Lund University and were intercrossed as described in **Figure 1A**. Rag1KO mice used as recipients were kindly provided by Dr. Joan Yuan, Lund University, and maintained by breeding at the CCM. All animal experiments were performed with approval from the Malmö/Lund animal ethics board (18055/2020 and M186-15).

**Figure 1:**
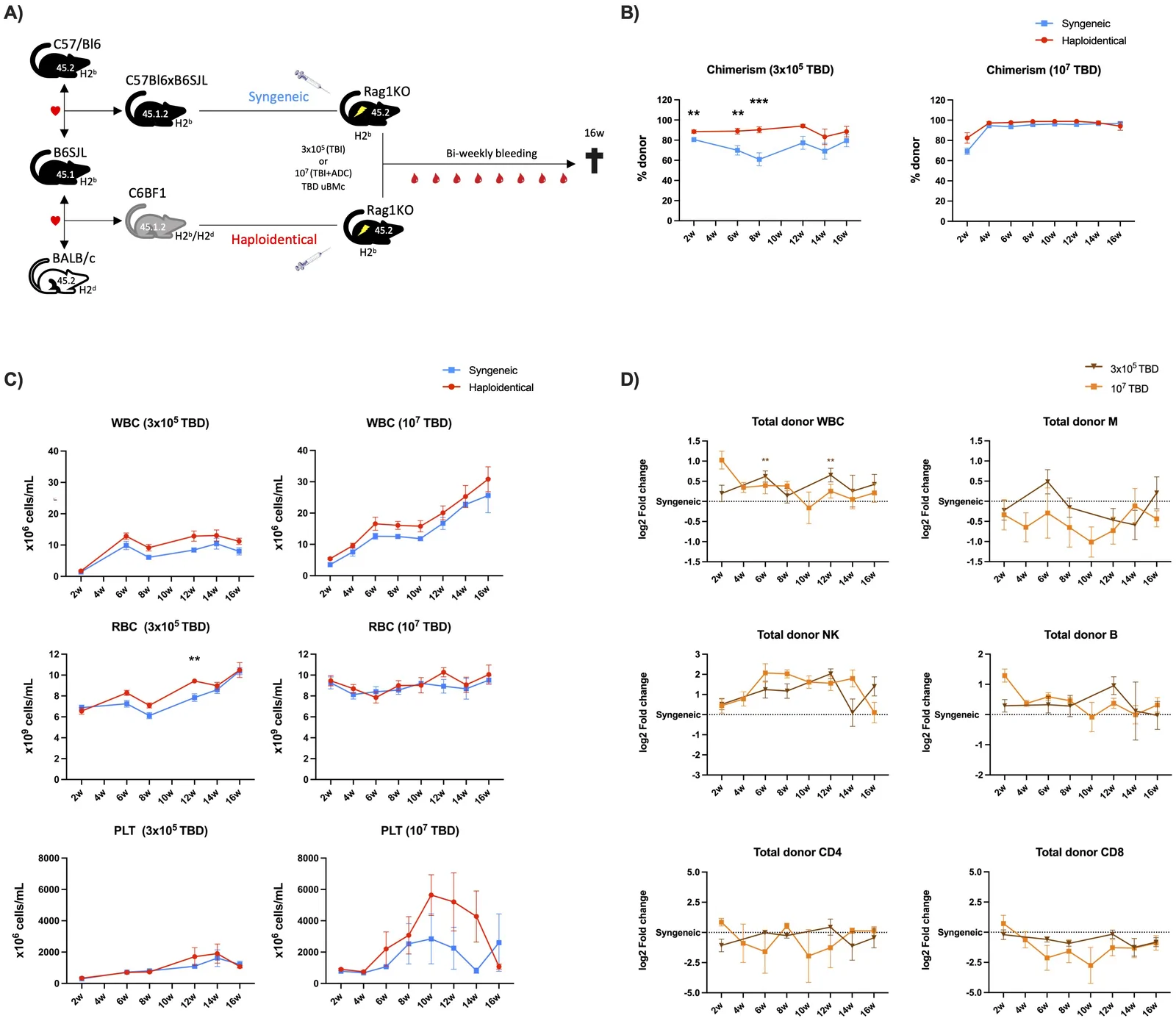
Haploidentical and identical TBD bone marrow cells achieve similar reconstitution in conditioned recipients. **(A)** Experimental design for the breeding strategy and transplantation of identical (C57Bl6×B6SJL) and haploidentical (C6BF1) donors (CD45.1.2) into previously conditioned Rag1KO recipients (CD45.2). Recipient mice that received 3×10^5^ T- and B-cell-depleted (TBD) bone marrow cells (uBMc) underwent conditioning with 800 cGy total body irradiation (TBI), and those receiving 10^7^ TBD BM cells were conditioned with sublethal TBI (200 cGy) plus 3 mg/kg of an antibody-drug conjugate (ADC), CD45.2-SAP. Mice were bled every two weeks and sacrificed 16 or 18 weeks after transplantation. **(B)** Total peripheral blood (PB) donor chimerism levels at the indicated times after transplantation, in the two experimental settings indicated for each graph. **(C)** Hematological counts of white blood cells (WBC), red blood cells (RBC), and platelets (PLT) at the indicated times after transplantation, in the two experimental settings indicated for each graph. **(D)** Normalized total numbers for each indicated cell type, with the corresponding mean value for the identically transplanted mice set to 1 for each experimental condition. Natural killer cells (NK), myeloid cells (M), B lymphocytes (B), CD4+ T cells (CD4), and CD8 T cells (CD8). Statistics indicate significance derived from comparison of the absolute values of the identical and haploidentical groups for each experimental setting. All data points indicate mean ± SEM. P-values were corrected for multiple comparisons using the two-stage Benjamini–Hochberg false-discovery-rate procedure and are reported as q-values (*q<0.05; **q<0.01; ***q<0.001). In transplantations with 3×10^5^ TBD cells, the initial n values were 27 for identical and 28 for haploidentical. In transplantations with 10^7^ TBD cells, the initial n values were 6 for identical and 7 for haploidentical.

### Mice transplantations

Recipient Rag1KO mice conditioned with lethal total body irradiation (TBI) alone (800 cGy) were transplanted 4–8 h after irradiation. Recipient Rag1KO mice conditioned with a combination of sublethal TBI (200 cGy) and immunotoxin received 3 mg/kg of CD45.2-SAP-conjugated antibody intravenously [prepared as previously described (23)] 8 days before transplantation. Sublethal irradiation was administered 4–8 h before the transplanted cells were given. Starting from the first conditioning intervention (lethal irradiation or immunotoxin treatment), mice received antibiotic prophylaxis in the drinking water for 2 weeks (ciprofloxacin, HEXAL, 125 mg/L).

Bone marrow cells from C57B6xB6SJL or C6BF1 donor mice were harvested by crushing all limb bones, pelleted, and stained with a master mix of biotinylated anti-mouse CD4 (clone GK1.5), CD8a (clone 53-6.7), and CD19 (clone 1D3) antibodies. Cells were then labeled with anti-biotin microbeads (Miltenyi Biotec), and positive cells were depleted on an AutoMACS instrument (Miltenyi Biotec) according to the manufacturer’s instructions. The corresponding number of T- and B-cell-depleted (TBD) cells (3x10^5^ or 10^7^) were administered intravenously through the tail vein in a final volume of 200 µL FACS buffer (PBS + 2% FBS + 2 mM EDTA) per mouse.

### Mice blood analysis

Following transplantation, mice underwent biweekly bleeding. Two to three drops of peripheral blood were applied directly onto a Guthrie card (PKI RUO Five Spot Card, PerkinElmer) for subsequent DNA extraction and ddPCR as described below. Next, up to 200 µL of peripheral blood was collected directly into EDTA-precoated Microvette tubes (Sarstedt), to be split and processed as follows:

Hematological counting: Cell counts were determined on a Sysmex KX-21N Automated Hematology Analyzer or a Sysmex XQ-320 (the same counter was used throughout a given experiment) after diluting the sample 1:2 or 1:5 in FACS buffer.

Plasma isolation: At least 20 µL of whole blood in EDTA tubes was centrifuged at 2000 × g for 5 min at room temperature (RT). The supernatant was collected and stored at -20 °C until use in the immunoglobulin or proinflammatory cytokine detection assays described below.

Flow cytometry: The remaining blood was transferred to tubes containing FACS buffer supplemented with heparin (LEO, 1:500). Erythrocytes were removed by incubation with Dextran T-500 (Sigma; 2% in PBS) at 37 °C for 30 min. The supernatants were pelleted, and the remaining red blood cells were lysed with ammonium chloride for 2 min at room temperature. Cells were stained for 30 min at 4 °C with the following conjugated anti-mouse antibodies: CD45.1-FITC (clone A20), CD8-PE-Cy5 (clone 53-6.7), CD19-PE-Cy7 (clone 1D3), CD44-PE (clone IM7), CD4-BV711 (clone RM4-5), CD11b-BV785 (clone M1/70), CD62L-BV421 (clone MEL-14), Gr1-APC-Fire750 (clone RB6-8C5), and Nkp46-AlexaFluor647 (clone 29A1.4). After staining, cells were resuspended in FACS buffer supplemented with propidium iodide (1:1000; Life Technologies) as a viability marker. Cells were analyzed on an LSR Fortessa (BD).

### Multiplexed ddPCR

DNA extraction: Guthrie cards containing blood spots were allowed to dry (dried blood spot, DBS) at room temperature (RT), protected from light, for between 3 h and overnight (o/n), and were then stored at -20 °C. After all samples from the same experiment had been collected, a single 3-mm² disk was punched from the center of each DBS and placed in a well of a 96-well plate. The DBSs were washed by pipetting with 100 µL of purification buffer (Generation DNA Purification Solution 1; Qiagen); the covered plate was centrifuged at 2000 rpm for 5 min, and the supernatant was discarded by pipetting. DNA was then eluted by incubation for 2 h at 50 °C in 25 µL of Tris-EDTA (pH 8) containing proteinase K (1:200) and 100 µg/mL yeast tRNA, followed by 10 min at 99 °C. The plate was centrifuged at 2000 rpm for 10 min, and the supernatants containing DNA were transferred to a new 96-well plate and stored at -20 °C until ddPCR was performed.

ddPCR: DNA from the DBS was mixed with ddPCR master mix (ddPCR Multiplex Supermix 4×;Bio-Rad), water, and primers and probes as indicated in **Supplementary Table 1**. The reaction mixtures and droplet generation oil (Bio-Rad) were processed in a QX200 droplet generator (Bio-Rad) according to the manufacturer’s instructions and then transferred to a 96-well semi-skirted ddPCR plate (Bio-Rad). The plate was sealed with foil in a plate sealer (Bio-Rad), and PCR amplification was performed in a C1000 Touch thermal cycler (Bio-Rad). The plate was stored overnight at 4 °C and read on a QX200 droplet reader (Bio-Rad) the following day. Data (absolute quantification) were acquired with QX Manager 1.2 Standard Edition (Bio-Rad).

### Immunoassays

Immunoassays for the detection of immunoglobulins in all transplantations and of proinflammatory cytokines after poly(I:C) treatment were performed on a MESO QuickPlex SQ 120MM reader (MSD) using the following Multi-Spot Assay Kits: the “Mouse Isotyping Panel 1 Kit” and the “V-PLEX Proinflammatory Panel 1 Mouse Kit” (MSD). In each case, plasma samples were diluted and prepared according to the manufacturer’s instructions.

### Flow cytometry with bone marrow and spleen

Bone marrow and spleens were manually homogenized into single-cell suspensions as previously reported (24). Cells were stained for 30 min at 4 °C with the indicated antibodies. The antibodies and clones used were: CD45.1 (clone A20), CD45.2 (clone 104), CD3e (clone 17A2), CD4 (clone GK1.5), CD8a (clone 53-6.7), CD19 (clone 1D3), CD11b (clone M1/70), B220 (clone RA-3-62), Gr1 (clone RB6-8C5), and Ter119 (clone Ter-119), together with streptavidin conjugated to several different fluorophores, and Nkp46-AlexaFluor647 (clone 29A1.4), cKIT-APCef780 (clone 2B8), Sca1-Pacific Blue (clone D7), CD150-APC (clone TC15-12F12.2), CD48-AlexaFluor700 (clone HM48-1), IgM-Brilliant Violet 421 (clone RMM-1), IgD-Brilliant Violet 605 (clone 11-26.2a), and CD24-FITC (clone M1/69). After staining, cells were resuspended in FACS buffer supplemented with propidium iodide (1:1000; Life Technologies) as a viability marker. Cells were sorted on FACSAria III or FACSAria IIu sorters and analyzed on LSR II or LSR Fortessa analyzers (all from Becton Dickinson). Mature cells and hematopoietic stem and progenitor cells were identified as previously described (24).

### Data collection and analysis software

Flow cytometry data were acquired with BD FACSDiva software and subsequently analyzed in FlowJo (TreeStar). Before FlowJo analysis, FACS files from peripheral blood analyses were curated and quality-controlled in FlowJo using the “flowAI” plugin (25). ddPCR data were obtained and analyzed with QuantaSoft software (Bio-Rad), and immunoassay data were collected by MSD Discovery Workbench (MSD). All data were statistically analyzed with GraphPad Prism v10 (GraphPad Software). Comparisons between the two donor groups (haploidentical versus syngeneic) were performed separately at each time point using unpaired Student’s t-tests on the absolute values; because the two groups comprise independent animals, each comparison uses a single, independent observation per animal, and no paired or across-time comparisons were made and the time factor was not formally modelled. Data are presented as mean ± SEM. To account for the multiplicity of comparisons made across the eight time points, p-values within each read-out were corrected across all time points using the two-stage Benjamini–Hochberg step-up procedure in GraphPad Prism and are reported as false-discovery-rate (FDR)-adjusted q-values; the significance indicated in the figures is based on these adjusted q-values. For the principal comparisons, effect estimates are additionally provided as the difference between group means (for absolute values) or the log2 fold change relative to the syngeneic mean (for normalized values), each with its 95% confidence interval (**Supplementary Table 3**). Emphasis is placed on differences that were consistent across multiple sequential time points and reproducible across the two independent transplantation settings.

### Experimental design and rigor

Transplantation experiments were performed in two independent cohort(s) for the 3×10^5^ setting and one independent cohort for the 10^7^ setting. Donor bone marrow was pooled from 2 + 2 and 1 + 1 donor mice in the 3x10^5^ setting experiments respectively and 3 + 3 mice in the 10^7^ cells setting before T- and B-cell depletion. Recipient mice were allocated to donor groups aiming for mice of difference groups being evenly hosted in the same cages. Blinded investigators were responsible for bleeding and sample processing and not blinded investigator during data acquisition and analysis. The experimental unit was the individual recipient mouse. Group sizes are stated in each figure legend; initial numbers were 27 (identical) and 28 (haploidentical) for 3×10^5^ TBD transplants and 6 (identical) and 7 (haploidentical) for 10^7^ TBD transplants. Number of animals included in each timepoint is indicated in **Supplementary Table 2** where decreasing numbers of analyzed data-points (compared to start numbers) are due to mice death, signs of temporary weakness before bleeding, or insufficient volume sample for all the test required. Reporting follows the ARRIVE 2.0 guidelines.

## Results

### Haploidentical TBD BM cells reconstitute the immune system at least as efficiently as syngeneic donor-derived cells

To assess the reconstitution capacity of haploidentical bone marrow donors relative to syngeneic (MHC-identical) donors in stem cell transplantation, we generated two F1 mouse strains by breeding WT B6SJL with WT C57Bl6 mice (C57Bl6×SJL (syngeneic): H2^b^, CD45.1.2) and WT BALB/c with WT B6SJL mice (C6BF1 (haploidentical): H2^b/d^, CD45.1.2). These two strains were used as bone marrow (BM) donors, while Rag1KO mice on a C57Bl6 background (H2^b^, CD45.2) were used as recipients following exposure to different conditioning regimens (**Figure 1A**). Because Rag1KO mice lack the ability to generate fully mature B or T cells, they provide a suitable system for studying the lymphoid output generated by the donor cells.

We performed two sets of transplantations: one in which we used 3×10^5^ T- and B-cell-depleted (TBD) BM cells after lethal total body irradiation (TBI; 800 cGy), and another in which 10^7^ TBD cells were transplanted after conditioning with a CD45.2 antibody-SAP conjugate (antibody-drug conjugate, ADC) combined with sublethal TBI (200 cGy).

In both experimental settings, we observed a tendency for the haploidentical cells to reach higher chimerism at an earlier time point than the syngeneic ones (**Figure 1B**). This effect was more pronounced in the 3×10^5^ cell setting, likely because 100% bone marrow reconstitution is reached sooner when 10^7^ cells are given to the recipient. To account for possible differences in the number of hematopoietic stem cells (HSC) or early progenitors between the two donor strains, we analyzed the content of HSCs, MPPs, and GMLPs in the donors used to transplant 10^7^ cells (**Supplementary Figure 2A**), as well as the total peripheral blood counts and subpopulations (**Supplementary Figures 2B, C**). These data revealed no major differences in steady-state primitive hematopoietic progenitors or in fully mature circulating blood cells.

Analysis of total blood cell counts also revealed higher total WBC (**Figure 1C** top) production when haploidentical cells were transplanted. This trend was not always significant but was consistent across all analyzed time points. Although no overall difference was detected in total RBC production (**Figure 1C** middle) in any of the experimental settings, we observed a slight advantage for the haploidentical donors over the syngeneic ones after transplanting 3×10^5^ cells; however, this difference appeared only within the 6-12-week window and returned to comparable levels at the latest time points. Although PLT production (**Figure 1C** bottom) tended to be higher in haploidentically transplanted mice than in syngeneic mice, no firm difference could be claimed because of the extremely high variability between mice after 10^7^ TBD BM cell transplantation, consistent with the very similar PLT output in the two groups after transplantation of 3×10^5^ cells.

After evaluating total chimerism and the overall hematological values, we further investigated the main peripheral blood cell populations by flow cytometry (**Supplementary Figure 1**). We tracked the different cell types derived from each donor type under both experimental conditions and normalized them to the total WBC count; the production of each cell type was then expressed relative to the mean production of the corresponding syngeneic counterparts (**Figure 1D**). As noted above, total WBC production was higher in haploidentical than in syngeneic recipients, and this difference was significantly more pronounced when 3×10^5^ cells were transplanted. Under both experimental conditions, this difference, particularly at the earliest time points, was linked to the consistently higher production of donor-derived NK cells in the haploidentical settings. This was a reproducible trend across time points and both experimental settings that did not, however, reach statistical significance after correction for multiple comparisons. A similar but weaker conclusion applies to B cell production, whose development kinetics were faster in haploidentically transplanted mice and remained higher than in syngeneic mice, though less markedly, at the remaining time points. By contrast, the haploidentical donors showed an overall tendency toward lower myeloid cell production under both conditions, except at one time point at which mice transplanted with 3×10^5^ cells exhibited more myeloid cells than their syngeneic controls. This difference later resolved, and the variability across all data points led us to conclude that there were no significant differences in this respect. A similar trend was observed for the CD4 and CD8 T cell compartments. Although one time point showed a small non-significant increase in CD4 cell production in the haploidentical compared with the syngeneic group under one of the conditions (3×10^5^), no other time points showed significant differences, indicating toward no significant differences in CD4 T cell production between the two groups. Similarly, CD8 T cells showed no significant difference in production between the two groups when 3×10^5^ cells were administered; however, a clear and consistent tendency toward fewer total CD8 T cells was seen at the intermediate time points (6–12 weeks) after 10^7^ cell transplantation. This tendency dissipated toward the end of the experiment, where CD8 T cells remained lower in the haploidentical than in the syngeneic settings but less markedly so.

### B cells show at least similar functional parameters in haploidentical and syngeneic transplant recipients

In addition to the quantitative evaluation of all WBC compartments in peripheral blood, we focused on the lymphoid output using both quantitative and functional approaches. For B cells specifically, we measured KREC (*kappa* recombination excision circles) production from dried blood spots (DBS) at every time point and measured the plasma concentrations of different immunoglobulin types. Although no differences in KREC production were detected at the earlier time points when 3×10^5^ TBD cells were transplanted (**Figure 2A** top), a trend towards KREC numbers in the haploidentically transplanted mice was observed at the later time points (8–12 weeks), which did not reach statistical significance after correction for multiple comparisons. These differences disappeared at the final time points, perhaps reflecting more rapid B cell division and the consequent dilution of KREC per cell number (%TCRA). In contrast, mice that received 10^7^ cells (**Figure 2A** bottom) showed no significant differences in KREC in both settings, with the greatest variability in both groups at the earliest time point.

**Figure 2:**
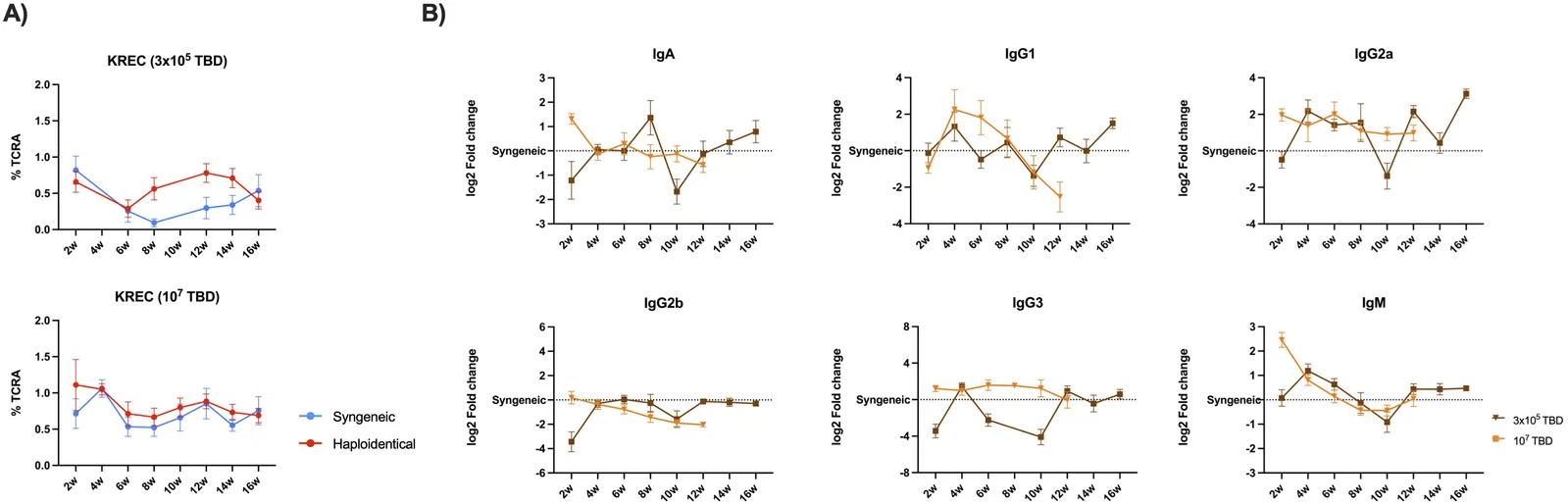
B cell functional parameters are equal or higher after haploidentical than after identical transplantation. **(A)** Kappa-recombination excision circles (KREC). Values are expressed as the percentage of positive droplets relative to positive droplets for the housekeeping gene (TCRA), determined by digital droplet PCR (ddPCR), in the two experimental settings indicated for each graph. **(B)** Normalized plasma concentrations (µg/mL) for each indicated Ig type, with the corresponding mean value for the identically transplanted mice set to 1 for each experimental setting. Statistics indicate significance derived from comparison of the absolute values of the identical and haploidentical groups for each experimental condition. All data points indicate mean ± SEM. P-values were corrected for multiple comparisons using the two-stage Benjamini–Hochberg false-discovery-rate procedure and are reported as q-values (*q<0.05; **q<0.01; ***q<0.001). In transplantations with 3×10^5^ TBD cells, the initial n values were 27 for identical and 28 for haploidentical. In transplantations with 10^7^ TBD cells, the initial n values were 6 for identical and 7 for haploidentical.

After measuring the plasma concentrations of six different immunoglobulin types, we observed heterogeneous results that depended largely on the number of cells initially transplanted (**Figure 2B**). When 10^7^ cells were transplanted, we observed faster antibody production kinetics, especially for IgA, IgG2a, and IgM, at the earliest time point (2 weeks) in the haploidentical donors; however, these differences were not detected with 3×10^5^ cell transplantation; for IgA and IgM, these higher values returned to levels equal to their syngeneic controls after 4 and 6 weeks, respectively. This upward tendency in the haploidentical group was maintained for IgG2a until the end of the experiment in both transplantation settings but began with a short delay in the 3×10^5^ transplanted mice. For IgG1 in mice receiving 10^7^ cells, the concentration reached its highest value relative to the syngeneic mice around the second time point (4 weeks) and then gradually decreased until it fell below the control between 8 and 10 weeks after transplantation. Up to this point, no significant differences were observed for IgG2b; thereafter, a lower (though after correction for multiple comparisons non-significant) concentration was seen when 10^7^ haploidentical cells were transplanted compared with the syngeneic transplanted group, which later returned to the levels of the syngeneic mice. Almost no significant differences in IgG3 concentration were detected between the syngeneic and haploidentical groups until weeks 14 and 16, when more changes occurred, with high variability.

For 3×10^5^ transplanted cells, IgA, IgG2b, and IgG3 all showed lower concentrations than the syngeneic controls at the first time point but reached or exceeded the syngeneic values after 4 weeks in every case and were thereafter generally maintained at similar levels. For IgG1 and IgM, with greater variability for IgG1, the levels were generally comparable to the control average at almost every analyzed time point.

To further investigate whether there were other important differences in B cell production and maturation, we performed FACS analysis of the donor-derived B cell progenitors in the bone marrow at 16 weeks (**Supplementary Figure 3A**). Analysis of absolute cell numbers showed no significant differences in the amounts of the different fractions between the two experimental settings. Comparable results were obtained for the absolute numbers of total B cells (CD19+) in the spleen of the recipient mice, with a slight but significant advantage of the haploidentically transplanted mice over the syngeneic mice that received 10^7^ TBD cells. (**Supplementary Figure 3B**).

### T-cell recombination shows no significant differences, whereas T-cell numbers differ in a compartment-specific manner, between the syngeneic and haploidentical transplantation settings

In the peripheral blood flow cytometry analysis (**Supplementary Figure 1**), we included the cell surface markers CD62L and CD44 to assess the activation state of the T cells, allowing us to distinguish naïve, effector, and central memory (CM) cells for each type (CD4 and CD8) (**Figure 3A**). These values were normalized to the total WBC count and, for each experimental setting, compared with the mean of the corresponding syngeneic controls (**Figure 3B**). Whereas almost no significant differences between groups were detected in the total numbers of the CD4 subsets after 3×10^5^ or 10^7^ transplantation, a strong tendency toward lower total cell production was apparent for all CD8 subcompartments in both haploidentical settings; this was most noticeable when 10^7^ cells were transplanted, especially for the CD8 effector cells, and for the CD8 CM cells when 3×10^5^ transplanted cells, after 12 weeks post-transplantation. To compare the recombination capacity of these T cells, we analyzed the relative amount of TREC (T-cell recombination excision circles) in the peripheral blood from the DBS obtained every other week after transplantation (**Figure 3C**). No significant differences were observed over time in any of the experimental settings, most likely because of the large variability within the groups.

**Figure 3:**
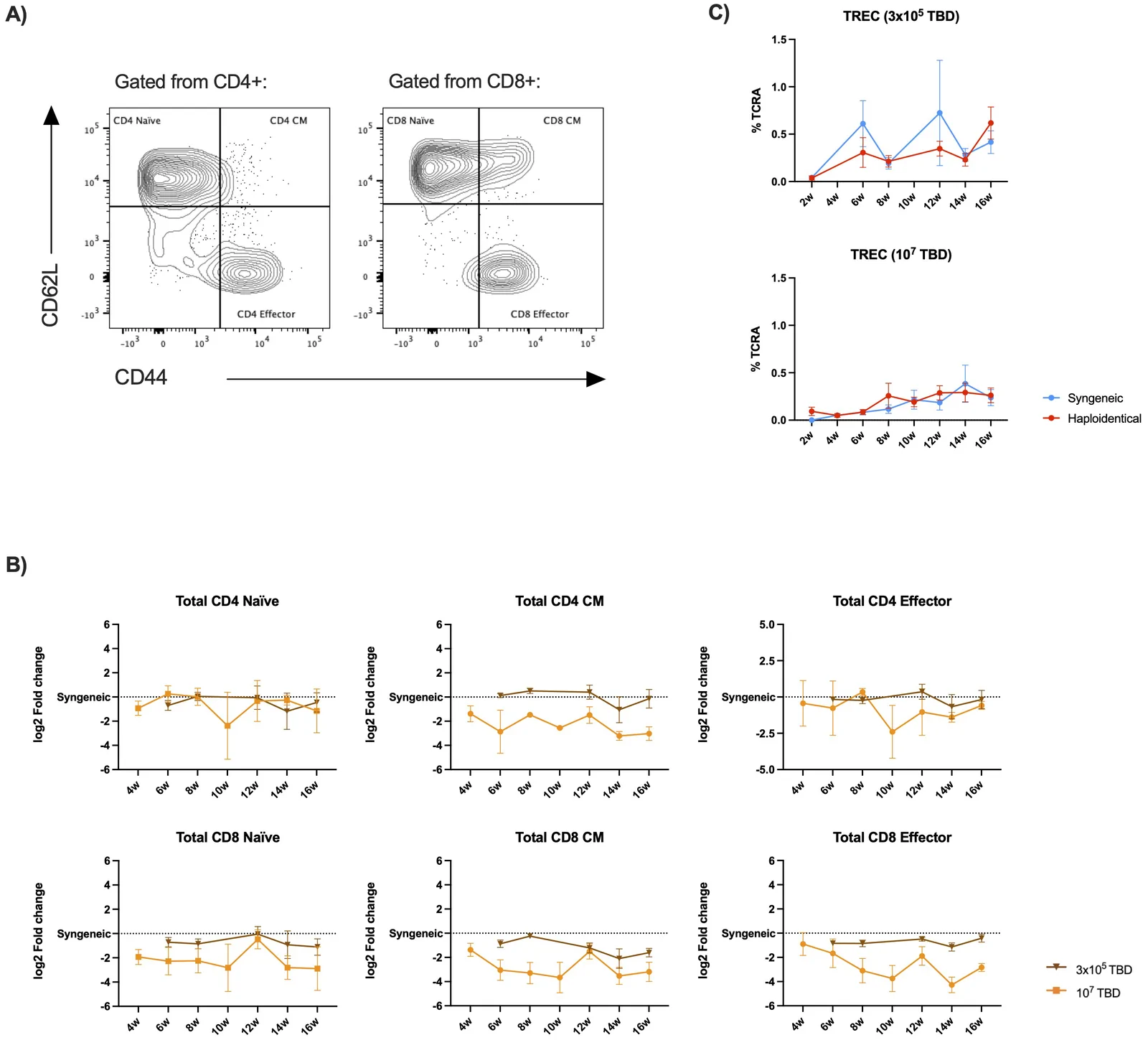
Haploidentically transplanted mice show lower T-cell-number reconstitution but equal functional-parameter kinetics compared with the identically transplanted group. **(A)** Gating strategy for the flow cytometry identification of naïve, effector, and central memory (CM) subsets of the CD4 and CD8 T cell populations. **(B)** Normalized total numbers for each indicated cell type, with the corresponding mean value for the identically transplanted mice set to 1 for each experimental setting. Statistics indicate significance derived from comparison of the absolute values of the identical and haploidentical groups for each experimental condition. **(C)** T-cell recombination excision circles (TREC). Values are expressed as the percentage of positive droplets relative to positive droplets for the housekeeping gene (TCRA), determined by digital droplet PCR (ddPCR), in the two experimental settings indicated for each graph. All data points indicate mean ± SEM. P-values were corrected for multiple comparisons using the two-stage Benjamini–Hochberg false-discovery-rate procedure and are reported as q-values (*q<0.05; **q<0.01; ***q<0.001). In transplantations with 3×10^5^ TBD cells, the initial n values were 27 for identical and 28 for haploidentical. In transplantations with 10^7^ TBD cells, the initial n values were 6 for identical and 7 for haploidentical.

### The inflammatory response to viral stimuli shows no significant differences between the syngeneic and haploidentical bone marrow transplantation settings

To functionally evaluate the inflammatory and immune response capacity of the newly reconstituted system against acute challenges such as viral infections, we generated chimeras in which Rag1KO mice receiving TBI were transplanted with a total of 10^7^ syngeneic or haploidentical TBD uBM cells (**Figure 4A**). After routine blood analysis at 8 weeks, when the mice already had a robust number of T cells in the peripheral blood, we challenged the mice 4 days later with intraperitoneal injections of 400 µg of poly(I:C), an analogue of double-stranded viral RNA. Peripheral blood was collected for flow cytometry, hematological counting, and plasma isolation 4 h after stimulation. The same procedure was performed 14 weeks after transplantation. For data analysis, we calculated, for each individual mouse, the values after the poly(I:C) injections relative to those at the immediately preceding bleeding, thereby assessing the changes induced by the poly(I:C) challenge. On hematological counting, we detected a decrease in the total number of donor-derived cells after both the 8- and 14-week injections (**Figure 4B**) relative to the previous time point, with no difference between the syngeneic and haploidentical groups. No differences were found in total RBC and PLT (**Supplementary Figure 4A**) counts at any time point, apart from a slight tendency for PLTs to be higher in the haploidentical group after the first challenge. Flow cytometry analysis of the main blood subpopulations, after normalization to total WBC counts, revealed no significant differences in the response between the haploidentical and syngeneic groups after each poly(I:C) injection (**Supplementary Figure 4B**), with a decrease in all WBC types except donor-derived myeloid cells compared with the saline controls. However, when we separately compared the changes relative to the time point preceding each injection, we found that after the first poly(I:C) challenge, the increase in myeloid cells was significantly greater in haploidentical than in syngeneic recipients (**Figure 4B**); these differences were absent after the second poly(I:C) injection at 14 weeks. To identify the populations mainly responsible for this increase, we fractionated the CD11b+ population according to their cellular complexity and CD11b expression level (**Figure 4D**) and observed that the main response after 8 weeks was an increase in neutrophils in both groups (**Figure 4C**); this increase was significantly greater under haploidentical than syngeneic conditions only after the first challenge.

**Figure 4:**
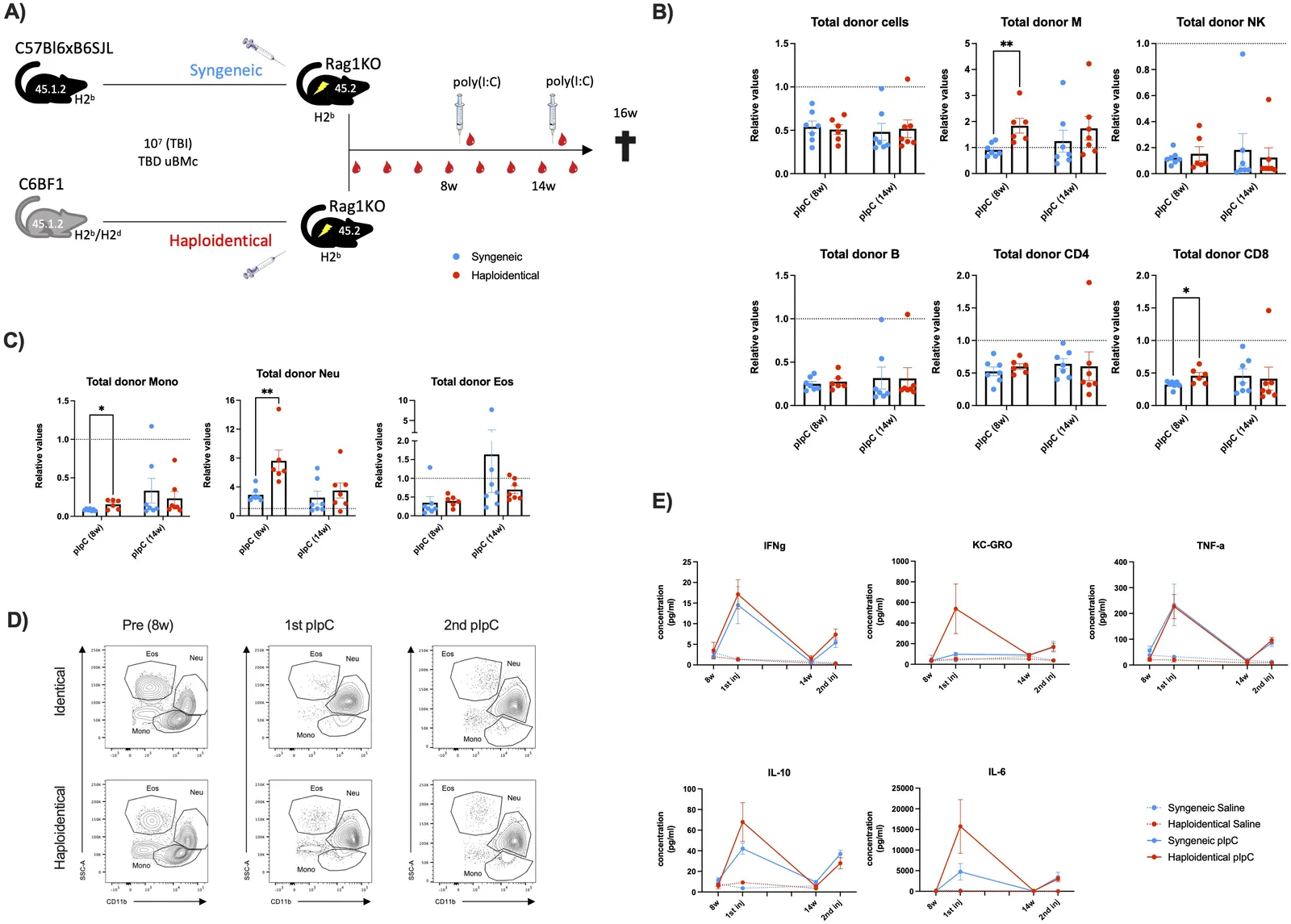
Similar inflammatory response to poly(I:C) stimulation in both transplantation settings. **(A)** Experimental strategy: Rag1KO mice received 800 cGy TBI and were transplanted with 10^7^ TBD uBMc from identical or haploidentical donors. Mice were bled every two weeks and sacrificed 16 weeks after transplantation (n = 9 + 9). At 8 weeks + 4 days and 14 weeks + 4 days, 7 mice from each group received an IP injection of 400 µg of poly(I:C), and the remaining 2 mice from each group received an equivalent volume of 0.9% NaCl solution (saline). All mice were bled 4 h after each injection and euthanized 16 weeks after transplantation. **(B)** Total donor-derived cells for each analyzed population. Dots represent values relative to the time point analyzed immediately before the indicated injection in the same individuals (=1). **(C)** Relative values for each myeloid population as a fraction of the total donor-derived cells. Dots represent values relative to the time point analyzed immediately before the indicated injection in the same individuals (=1). **(D)** Gating strategy for the flow cytometry identification of monocytes (Mono), neutrophils (Neu), and eosinophils (Eos) within previously gated myeloid cells. All bars indicate mean ± SEM and dots represent individual values. *p<0.05; **p<0.01; ***p<0.001. n = 7 individuals per group. **(E)** Mean cytokine concentrations before and after the first (8w) and second (14w) injections of poly(I:C) or saline. Dots and error bars indicate mean ± SEM. n = 7 individuals per group for poly(I:C) and 2 per group for saline.

Plasma collected before and after the two poly(I:C) injections was analyzed by MSD immunoassay to measure the levels of the most common proinflammatory cytokines (**Figure 4E**). In both transplantation types, we detected an increase in IFN-γ, IL-10, and TNF-α relative to the saline controls after the first injection; however, a more noticeable increase in KC-GRO, and to a lesser extent in IL-6, was reached in the plasma of the haploidentical than the syngeneic group. These changes had resolved by week 14, when all affected cytokines had returned to basal levels. The second poly(I:C) challenge produced a much milder increase in all the previously altered cytokines in both groups, indicating no significant difference in the response to the virus-mimicking stimulus between the haploidentical and syngeneic transplanted mice.

## Discussion

We have developed a system that allows detailed characterization of the immune reconstitution mediated by the donor cells in hematopoietic cell transplantation, without interference from residual recipient lymphoid cells. A similar experimental setup was reported and successfully demonstrated the capacity of haploidentical cells to correct Rag1 deficiency (26); however, we focused on a direct comparison of syngeneic and haploidentical engraftment and lymphoid reconstitution from both quantitative and functional perspectives. Our initial findings revealed a higher engraftment capacity of the haploidentical than the syngeneic donor cells, which was more pronounced at the lower cell dose, whereas both groups reached a plateau when 10^7^ cells were transplanted. A possible explanation for this advantage, consistent with our results, is the higher total number of NK cells in the haploidentical than in the syngeneic transplanted group. Previous studies have described mechanisms by which recipient NK cells (C6BF1) can reject parental bone marrow-derived cells on the basis of a balance between different activating and inhibitory signals (27), including “missing-self recognition” among the former, in haploidentical settings (C6BF1 recipients transplanted with C57Bl6 or BALB/c cells). In our case, although in the opposite direction, this could potentially explain a partial activation of the donor-derived NK cells, leading to their expansion without overt signs of GvHD; although NK-cell signaling can contribute to inflammation and tissue injury, GvHD itself is not primarily NK-cell-mediated. Comparable results were shown in an immunocompetent mouse model in which F1 cells were transplanted into a parental recipient and their alloreactivity allowed the omission of an additional conditioning regimen by causing immune ablation but not GvHD (28). Other murine haploidentical models have been used mainly to dissect graft-versus-host disease and its prevention rather than immune reconstitution; for example, in a T-cell-replete MHC-haploidentical model, post-transplant cyclophosphamide was shown to prevent graft-versus-host disease primarily by inducing functional impairment of alloreactive T cells and preferential recovery of regulatory T cells, rather than by deleting them (29). In contrast, our T- and B-cell-depleted, Rag1-deficient system is free of graft-versus-host disease and isolates the reconstitution derived from the donor cells.

Our data revealed no significant differences in *de novo* B cell and immunoglobulin production between the two transplantation types. Notably, IgG2a levels were higher in recipients of haploidentical cells. However, reduced IgG2a levels have been reported in the C57Bl6 strain (30), so these differences likely reflect strain variability rather than the syngeneic or haploidentical nature of the donor cells. These findings are consistent with previous studies in patients showing that factors such as donor or recipient age, but not donor type (among other factors), affect B cell reconstitution in the recipient (31). More recently, the same has been observed for overall survival (32). Regarding *de novo* T cell production, we did not observe significant differences in TREC/cell concentrations or in naïve CD4 or CD8 T cells; however, lower values for CD8 effector cells were commonly seen in the haploidentical settings, probably accounting for the lower absolute numbers detected in the overall CD8 cell pool. Comparable results have been observed in patients, although without graft manipulation, in a cohort study in which the haploidentical group showed faster NK cell repopulation, slower T cell appearance, and no effect on B cell kinetics (33).

Because viral infections and reactivations are among the greatest challenges in haploidentically transplanted patients (33, 34), we assessed the functionality of the newly reconstituted immune cells using poly(I:C) injections. As an analogue of double-stranded RNA, poly(I:C) is a strong inducer of the type I interferon (IFNα/β) responses through activation of TLR3 (35, 36) but also independently through MDA5 (37). The first mechanism has been shown to be essential for the CD8 effector cell response, whereas the second is required for CD8 T cell memory in the presence of a specific antigen (38). In the absence of a specific antigen, we observed no significant differences in the proinflammatory effects between the two transplantation types, with a more pronounced increase in neutrophils in the haploidentical than in the syngeneic recipient mice after the first injection, a finding that correlates with the noticeably, though not significantly, higher plasma concentration of KC-GRO (CXCL1), which plays a key role in the recruitment and activation of neutrophils (39). Although CXCL1 is considered a proinflammatory cytokine, recent studies incorporating human umbilical cord-derived mesenchymal stem cells (hUCMSCs) into fully mismatched HCT have demonstrated an important regulatory role in the prevention of GvHD, associated with the high CXCL1 secretion levels of these cells (40). Its significance in the context of haploidentical transplantation, and the reason for its greater abundance in the haploidentically transplanted mice, require further investigation. The same tendency was found for IL-6 concentrations. High IL-6 levels have been associated with poor prognosis and GvHD in haploidentically transplanted patients (41), with improved outcomes following anti-IL-6 therapy to mitigate the effects of cytokine-release syndrome (CRS) (42); however, these studies were carried out in T-cell-replete transplant recipients. Therefore, a detailed exploration of inflammatory cytokines and viral responses in haploidentical and syngeneic T- and B-cell-depleted transplantation settings remains to be performed.

Several limitations of this study should be acknowledged. First, because the haploidentical (C6BF1) and syngeneic (C57Bl6×B6SJL) grafts originate from genetically distinct donor backgrounds, MHC disparity could confound with broader donor-strain differences. Consequently, phenotypes such as the higher NK-cell output, or differences in individual immunoglobulin subclasses such as IgG2a, cannot automatically be attributed to haploidentity itself, as already noted for strain-dependent IgG2a expression. Second, the Rag1-deficient recipient lacks endogenous mature B and T cells, which is advantageous for isolating donor-derived lymphopoiesis but does not recapitulate the residual host immunity and host–graft interactions present in most clinical transplants. Third, the T- and B-cell-depleted, Rag1-deficient design is by construction free of graft-versus-host disease, so our conclusions concern immune reconstitution rather than GvHD or graft-versus-tumor effects. Fourth, some experiments—particularly the antibody-drug-conjugate/10^7^-cell setting—used small group sizes (6–7 animals) with substantial inter-animal variability, which limits statistical power; the absence of a statistically significant difference in these comparisons should therefore not be interpreted as evidence of equivalence, since the study was not prospectively designed or powered as a non-inferiority trial. Finally, functional immune competence was probed with a single viral-mimic (poly(I:C)) challenge and did not include live-pathogen infection or antigen-specific recall responses, so the functional characterization remains limited in scope. These considerations should be taken into account when extrapolating our findings to the clinical setting.

Our study provides a comprehensive approach that sheds light on immune reconstitution in these two settings, which cannot be directly compared in the clinic. We demonstrate here that the haploidentical nature of the transplanted cells does not directly affect the cellular repopulation of the recipient mice. This substantially supports considerations of increasing the use of this type of donor and opens the field to further investigation of their potential therapeutic advantages. From a clinical perspective, the choice of donor remains a central decision in the management of HCT. When neither a matched sibling nor a MUD is available, alternative donors such as haploidentical donors are required, and in some cases the choice lies between a sibling/MUD and a haploidentical donor (43, 44). Haploidentical HCT is associated with certain clinical disadvantages, one of the most important being a delayed immunologic reconstitution and the accompanying infectious risk. Clinically, it has remained unclear whether this delay reflects the haploidentical nature of the graft itself or is instead driven by the more intensive conditioning and more extensive graft manipulation that these transplants typically require (14, 15). Because these variables are confounded in patients and cannot readily be separated in clinical studies, controlled murine models such as the one presented here are needed to isolate the effect of donor type. Our body of work indicates that, at least in this model, it does not appear to be the haploidentical nature of the cells that underlies a delayed immunologic reconstitution, supporting the view that the slower recovery observed in the clinical setting is more likely explained by conditioning and graft-manipulation factors than by cell-intrinsic properties of haploidentical grafts. Consistent with this interpretation, recent analyses of immune reconstitution after haploidentical HCT indicate that reconstitution kinetics are shaped substantially by the conditioning regimen and graft-versus-host disease prophylaxis strategy rather than by donor haploidentity alone (45). Beyond its clinical implications, this donor-only Rag1-deficient system offers a tractable platform for mechanistic studies of hematopoietic reconstitution — including NK-cell-mediated engraftment, innate responses to viral mimics, and the recovery kinetics of B and T cells — in the absence of graft-versus-host disease.

## Data Availability

The original contributions presented in the study are included in the article/**Supplementary Material**. Further inquiries can be directed to the corresponding author/s.

## Supplementary material

Supplementary Figure 1 Representative FACS contour plots showing the gating strategy used for all peripheral blood analyses.

Supplementary Figure 2 Direct comparison of the absolute counts of the indicated WBC types (cells/mL) between the two strains used for the transplantation experiments. **(A)** Total counts of hematopoietic stem cells (HSCs), multipotent progenitors (MPPs), and granulocyte-monocyte-lymphocyte progenitors (GMLPs) in the bone marrow. **(B)** Hematological counts of WBC, RBC, and PLT in the peripheral blood of the two indicated strains. **(C)** Absolute counts of the FACS-analyzed populations in the peripheral blood of the two mouse strains used. Dots indicate individual values; bars indicate the mean, and error bars indicate SEM. *p<0.05. n = 3 individuals per group.

Supplementary Figure 3 **(A)** Absolute values for the B cell progenitor subpopulations (Hardy fractions) analyzed by FACS in the bone marrow (BM) for each of the indicated transplantation conditions 16 weeks after transplantation. **(B)** Absolute values for mature B cells (CD19+) analyzed in the spleen for each of the indicated transplantation conditions 16 weeks after transplantation. Dots indicate individual values; bars indicate the mean, and error bars indicate SEM. *p<0.05. In transplantations with 3×10^5^ TBD cells, the n values were 13 for identical and 15 for haploidentical. In transplantations with 10^7^ TBD cells, the n values were 5 for identical and 6 for haploidentical.

Supplementary Figure 4 **(A)** Hematological counts of RBC and PLT relative to the paired value for each mouse at the corresponding time point before the indicated injection (=1). Bars indicate mean ± SEM, and dots represent individual values. **(B)** Absolute values for all donor-derived populations analyzed in the peripheral blood before and after each poly(I:C) or saline injection. Dots indicate the mean, and error bars indicate SEM. n = 7 individuals per group for poly(I:C) and 2 per group for saline.

Supplementary Table 1 Overview primer and probes for the ddPCR experiments.

Supplementary Table 2 Number of animals included in the peripheral blood analysis at each time point.
